# ASO Author Reflections: The Treatment Challenge of cT2cN0 Staged Adenocarcinoma of the Esophagus and the Gastroesophageal Junction

**DOI:** 10.1245/s10434-025-18520-1

**Published:** 2025-10-21

**Authors:** Naita M. Wirsik, Thomas Schmidt, Christiane J. Bruns

**Affiliations:** Department of General, Visceral, Thoracic and Transplant Surgery, University Hospital of Cologne, Cologne, Germany

## Past

The question whether cT2 cN0 staged adenocarcinoma of the esophagus and gastroesophageal junction should be treated with a multimodal approach remains unanswered owing to the lack of randomized controlled trials.^1^ Nonetheless, a multicenter European cohort analysis showed that even in high-volume centers approximately one-third of cT2 patients are still understaged and therefore do not receive the appropriate oncologic treatment.^2^ Furthermore, since the results of the ESOPEC trial have been published^3^ the treatment modality for adenocarcinoma of the esophagus has been adjusted to a FLOT based multimodal treatment in Europe.^4^

## Present

Our study analyzed the impact of a multimodal treatment for a specific cohort of cT2 cN0 staged patients with radio-chemotherapy according to the CROSS protocol in comparison with the FLOT protocol. We demonstrated that treatment with FLOT resulted in a survival benefit and was an independent factor for overall survival in the multivariable analysis. In summary, the multimodal treatment according to the FLOT protocol should be considered the preferred approach for cT2cN0 staged esophageal adenocarcinoma.^5^

## Future

The main issue remains how to adequately stage this specific subgroup of patients. Artificial intelligence may play a fundamental role in the future. However, at present, multimodal treatment with FLOT should be considered for cT2 staged patients as understaging could significantly worsen the prognosis of the patient. In addition, artificial intelligence could support the identification of patients that will benefit from a specific pretreatment based on the tumor biology in future.

## References

[1] Donlon NE, Moran B, Kamilli A, et al. CROSS Versus FLOT regimens in esophageal and esophagogastric junction adenocarcinoma: a propensity-matched comparison. *Ann Surg*. 2022;276(5):792–8. 10.1097/SLA.0000000000005617. 35876385 10.1097/SLA.0000000000005617

[2] Wirsik NM, Kooij CD, Dempster N, et al. Optimal treatment strategies for cT2 staged adenocarcinoma of the esophagus and the gastroesophageal junction: a multinational, high-volume center retrospective cohort analysis. *Ann Surg*. 2024;280(5):799–807. 10.1097/SLA.000000000000647. 39109441 10.1097/SLA.0000000000006478

[3] Hoeppner J, Brunner T, Schmoor C, et al. Perioperative chemotherapy or preoperative chemoradiotherapy in esophageal cancer. *N Engl J Med*. 2025;392(4):323–35. 10.1056/NEJMoa2409408. 39842010 10.1056/NEJMoa2409408

[4] Obermannová RL, Leong T; ESMO Guidelines Committee. Electronic address: clinicalguidelines@esmo.org. ESMO Clinical Practice Guideline interim update on the treatment of locally advanced oesophageal and oesophagogastric junction adenocarcinoma and metastatic squamous-cell carcinoma. *ESMO Open*. 2025;10(2):104134. 10.1016/j.esmoop.2025.104134. 10.1016/j.esmoop.2025.104134 PMC11889489 39986705

[5] Wirsik NM, Schmidt T, Kooij CD, et al. Comparison of pretreatment strategies for cT2cN0 staged adenocarcinoma of the esophagus and the gastroesophageal junction: a European high-volume center cohort analysis. *Ann Surg Oncol*. 2025. 10.1245/s10434-025-18311-8. 41083831 10.1245/s10434-025-18311-8 PMC12589201

